# Measuring and tracking obesity inequality in the United States: evidence from NHANES, 1971-2014

**DOI:** 10.1186/s12963-016-0081-5

**Published:** 2016-04-04

**Authors:** Tae-Young Pak, Susana Ferreira, Gregory Colson

**Affiliations:** 1 Department of Financial Planning, Housing, and Consumer Economics, University of Georgia, 205 Dawson Hall, 305 Sanford Drive, Athens, GA 30602 USA; 2 Department of Agricultural and Applied Economics, University of Georgia, 314-D Conner Hall, 147 Cedar Street, Athens, GA 30602 USA; 3 Department of Agricultural and Applied Economics, University of Georgia, 314-E Conner Hall, 147 Cedar Street, Athens, GA 30602 USA

**Keywords:** BMI distribution, Obesity inequality, Stochastic dominance, Shapley decomposition, Gini coefficient

## Abstract

**Background:**

Because people care about their weight relative to peers and society, obesity inequality plays a role in explaining obesity incidence and the impacts of being obese on subjective well-being. While the increase in obesity prevalence and mean body mass index (BMI) is well documented, the measurement of distributional changes and corresponding obesity inequality is yet to be fully explored.

**Methods:**

The present study analyzed BMI data for adults aged 20 to 74 from the National Health and Nutritional Examination Survey (NHANES) I (1971-1974), II (1976-1980), III (1988-1994), and continuous NHANES (1999-2014). We applied tools developed to measure income inequality to analyze the inter-temporal variation in the BMI distribution among US adults. Using stochastic dominance tests, we construct partial orderings on cumulative BMI distributions during the study period. Shapley decompositions and inequality indices are employed to quantify the source and extent of temporal variation and decompose the inequality into within and between-group components considering age, gender, and race.

**Results:**

The BMI distribution of each NHANES study first-order stochastically dominated the BMI distribution of the previous wave from 1971-1974 to 2003-2006, whereas more recent comparisons failed to reject the null hypothesis of non-dominance. The Shapley decomposition analysis revealed that horizontal shifts of BMI distributions accounted for a majority of the increase in obesity prevalence since 1988-1991. Especially in recent years when the rate of obesity growth has slowed down, the contribution of the redistribution component dropped significantly and even became negative between 2007-2010 and 2011-2014. The inequality indexes consistently show a worsening of obesity inequality from the mid-1970s to the mid-2000s regardless of population subgroups, and this disproportionate shift of the BMI distribution is unlikely to be a result of a changing ethnic composition of the US population.

**Conclusion:**

Our findings demonstrate that seemingly similar increases in obesity prevalence can be accompanied by very different patterns of distribution change. We find that the early phase of the obesity epidemic in the US was largely driven by increasing skewness, whereas more recent growth is a population-wide experience, regardless of demographic characteristics. Increasing morbid obesity certainly played an important role in the initial phase of the epidemic, but more recently the BMI distribution has largely horizontally shifted to the right.

## Background

Many studies have documented a marked increase in obesity prevalence and mean body mass index (BMI) in the US over the last four decades [1–6]. This significant and consistent rise in bodyweight has been termed an "obesity epidemic," spreading across all gender, age, and ethnic groups. The use of such language evokes the idea of obesity being contagious, spreading from one person to another. For instance, gains in weight appear to spread through social ties, with friends and relatives apparently influencing others in their social network, in a way reminiscent of a contagious disease [7].

In conjunction with an overall rise in obesity prevalence, there has been an even more significant increase in the percentage of US adolescents [8, 9] and adults [4, 10, 11] who are morbidly obese. This is reflected in a rightward shift of the BMI distribution, more pronounced at its upper tail [12]. Such changes in the shape of the BMI distribution, and in particular the disproportionate growth in the distribution’s upper tail, have been explained by models that interact the effects of economic change (e.g., falling food prices) with social and physiological processes. In the social process, a person’s body weight standard depends on other people’s weight, and a relaxed standard can lead to weight increases [13–15]. In a society where one’s weight does not conform to the socially ideal weight, social pressure may exist, and result in a disutility cost to individuals [16].

In a study of 29 European countries, evidence suggests that overweight perceptions and dieting are influenced by a person’s relative BMI [17]. The authors suggest that, for a variety of reasons, it may be easier to be fat in a society that is fat, and provide empirical evidence that relative BMI influences subjective well-being. A more recent study demonstrates that the degree to which obesity is negatively associated with life satisfaction can be mitigated by the prevalence of obesity in a given geographic context [18].

Despite the importance of relative obesity in explaining the causes and consequences of the obesity epidemic, little is known about the evolution of BMI distributions over time. A notable exception is Contoyannis and Wildman in which relative distribution methods are employed to track changes in BMI using nonparametric methods [19]. Focusing on Canada and England, they found that the increase of obesity in England is characterized by more polarized growth towards the right-end of the BMI distribution, whereas the increase of obesity in Canada is driven primarily by an overall upward shift. While growing obesity inequality is believed to be a population-wide experience [12], a recent study of Americans (1999-2006) revealed a different pattern of polarization, with a more pronounced shift among ethnic minorities and the less educated [20].

Building upon this work, we expand on the focus, methods, and time horizon of earlier studies to present a long-term picture (1971-2014) of obesity inequality in the US incorporating a wide array of quantitative methods used in the study of income inequality [21, 22]. In addition to presenting an encompassing measurement of the transition of BMI inequality during the US obesity epidemic, the economic tools employed to analyze changes in obesity offer several new insights.

First, borrowing tools common in the study of poverty, we employ Stochastic Dominance (SD) tests. SD is very useful when making non-parametric comparisons between distributions of continuous variables such as income or, in our case, BMI. SD tests offer an ordinal comparison between distributions; a comparison that ranks the distributions but that does not estimate the magnitude of the differences between them. Because SD tests involve comparison over the BMI domain, they are independent of the choice of an obesity threshold. Moreover, as we explain below, the computation of statistics of dominance at multiple test points covering the whole range of the BMI distributions enables us to assess whether the shift of the BMI distribution was driven primarily by one part of the distribution or by the entire population [21, 22]. Second, we apply the Shapley-value based decomposition technique to decompose total change in obesity prevalence into a mean-growth effect and a redistribution component [21, 23, 24]. While the SD test primarily focuses on determining the ordinal dominance of BMI distributions, this second approach shows how much of the growth in obesity inequality is attributable to a horizontal shift of the distribution (an increase in average BMI), and how much is due to a change in the shape of the distribution (in particular, an increased skewness towards the right tail of the distribution). Third, we provide a single-value quantitative assessment of the degree of inequality measured by conventional inequality indices (Gini and Generalized Entropy). When analyzing univariate inequality measures, we pay particular attention to decomposing obesity inequality into inequalities within each segment of the population and inequalities between subgroups. This complementary approach allows us to reveal detailed aspects of the transition across subpopulations and examine whether a disproportionate growth of obesity is due to population-wide shifts of the BMI distribution or to a changing contribution by the different demographic groups.

Several studies point out that focusing on prevalence estimates is at best a crude approach to understanding the obesity epidemic since it ignores much of the available information, and the measurement of obesity rates depends heavily on somewhat arbitrary thresholds and does not correctly reflect the clinical implications of obesity around cutoff values [19, 21, 22, 25, 26]. Since the BMI distribution in the US shows that most individuals are centered around the overweight category (i.e., a BMI greater than or equal to 25), even a minor rightward shift of the BMI distribution would result in significantly higher prevalence estimates, which might overestimate the seriousness of obesity. If the recent growth of BMI is more pronounced for those at the right tail of the distribution, tracking only prevalence estimates over time does not correctly reflect accompanying mortality and morbidity risks. Complementing obesity prevalence estimates with distribution-independent techniques such as those proposed by this paper is thus critical to understanding the long-term pattern of obesity change in the US.

## Methods

### Data sources and study population

Baseline data were drawn from the National Health and Nutritional Examination Survey (NHANES) I (1971-1974), II (1976-1980), III (1988-1994), and continuous NHANES (1999-2014). The NHANES is a series of nationally representative cross-sectional surveys of the US population, conducted by the National Center for Health Statistics at the Centers for Disease Control and Prevention. The NHANES data include clinical measurements of the respondents’ height and weight obtained using mobile examination centers and standardized procedures. This is an attractive feature of the data because self-reports of height and weight tend to be biased, leading to an underestimate of BMI [27–29]. This is particularly important when observations are clustered around the middle of the distribution since individuals whose BMI is close to the obesity threshold are more likely to under-report their weight [21].

For this study, we restricted the analysis to 20-74 year olds because only persons aged 1-74 years were eligible to be interviewed for NHANES I and II, and different weight classification criteria are used for people under 20. Following NHANES analytic guidelines [30], respondents were classified into three age groups, 20-39 years, 40-59 years, and 60-74 years, based on age at the examination date. For NHANES III and continuous NHANES, race and ethnicity were classified as non-Hispanic white, non-Hispanic black, Hispanic, and other. To facilitate comparability across waves, given the larger number of years covered by the initial waves, we aggregated two adjacent waves of continuous NHANES into a single survey. Since NHANES III covered a six year period (1988 to 1994) when the largest increase in obesity was experienced, we split NHANES III into two different periods, Phase I (1988-1991) and Phase II (1991-1994), according to NHANES III analytic guidelines [31]. The multistage sampling design of NHANES III selects 81 primary sampling units for a full six year survey, and then randomly assigns each primary sampling units to Phase I and Phase II, which makes each subsample representative of the US civilian non-institutionalized population during the given period. In addition, we excluded female respondents who were pregnant at the time of the survey. The final sample for empirical analysis did not include observations with missing values or irregular responses in height and weight.

We adopted the clinical definition of obesity proposed by the International Obesity Task Force of the World Health Organization in 1997. Underweight is defined as BMI < 18.5, normal weight as BMI in the interval [18.5, 25.0), overweight as BMI ∈[25.0, 30.0), class I obesity as BMI ∈[30.0, 35.0), class II obesity as BMI ∈[35.0, 40.0), and class III obesity as BMI ≥40.0. The prevalence or increase in BMI of morbid or clinically severe obesity across waves was of special interest to this research since many direct medical costs associated with obesity are most pronounced for those at the higher spectrums of the obesity scale.

To account for the complex, stratified, multistage probability cluster sampling design of NHANES, we applied mobile examination centers sampling weights throughout the analysis. For NHANES III, a 3-year sampling weight was applied to each Phase I and II, which benchmarked the 1990 and 1993 Current Population Survey (CPS), respectively [31, 32]. For the first combined sample of continuous NHANES, we used four-year sample weights for 1999-2002, which was pre-adjusted by NHANES to account for the difference in the population base of the 1999-2000 and 2001-2002 surveys [30]. By rescaling a two-year weight of adjacent surveys, this sample weight allowed us to make our sample representative of the population at the midpoint of the two surveys, even if different population bases were considered. For the subsequent four-year datasets, we created a four-year sample weight variable that assigned half of the two-year weight for each period, as recommended by NHANES analytic guidelines [30]. We could then compare the distributions over time, since these weighting schemes were designed to ensure that the weighted sample was representative of the US civilian non-institutionalized population; that is, it reflected the relative proportion of each demographic group to ensure equal selection probability of an individual given that some groups were oversampled.

### Stochastic dominance test

The Stochastic Dominance test is an approach that allows an ordinal assessment of whether a cumulative distribution significantly differs from another without considering the shape of the distribution [33]. We applied the test here to determine the dominance of BMI distributions of US adults over time. Although commonly used by economists in poverty and economic inequality studies [33–35], it has only recently been applied to the study of obesity [21, 22].

Let ${F}_{t_{n-1}}(x)$ and ${F}_{t_n}(x)$ denote two cumulative distribution functions (CDF) of BMI to be compared to each other, where *t*_*n-1*_ and *t*_*n*_ refer to time, i.e. to different NHANES waves, and${D}_t^1(x)={F}_t(x)={\displaystyle \underset{0}{\overset{x}{\int }}d{F}_t(y)}$and${D}_t^s(x)={\displaystyle \underset{0}{\overset{x}{\int }}{D}_t^{s-1}(y) dy}$ for any integer *s* ≥ 2. The distribution at time *t*_*n *_dominates the distribution *t*_*n* − 1 _at order *s* if ${D}_{t_{n-1}}^s(x)\ge {D}_{t_n}^s(x)$ and strictly dominates if ${D}_{t_{n-1}}^s(x)>{D}_{t_n}^s(x)$, for all possible BMI values over the domain [21, 33].

Simple *t*-statistics were constructed to test the null hypothesis of non-dominance (${H}_0:\kern1em {D}_{t_{n-1}}^s(x)-{D}_{t_n}^s(x)=0$), for a series of test points up to the maximum BMI in the distribution. Unlike other studies testing dominance only within a range of interest, we tested the significance over the entire domain (we used 30 test points from the minimum to the maximum BMI) in order to investigate which part of the distribution changed most. Dominance of order *s* was declared if the null hypothesis was rejected for at least one test point at the 1 % significance level without any reversal in the signs of difference [21]. The stochastic dominance does not hold if, for instance, the difference is not significant or two cumulative distributions cross each other. In general, it has been shown that the stochastic dominance of one distribution over another can always be declared at a high enough order, provided that infinite comparisons between CDFs can be made [37]. The interpretation of higher-order comparisons is, however, less intuitive [38] and, in practice, comparisons are limited to third-order stochastic dominance [33]. We followed the convention of testing up to *s* = 3, i.e. third-order stochastic dominance, after which "no dominance" is declared [22, 35, 36].

### Growth-inequality decomposition

The growth-inequality decomposition method allows researchers to decompose overall changes in a distribution into a mean-growth component and a redistribution component [39]. In the context of obesity, the mean-growth component captures the change in obesity prevalence attributable to a horizontal shift of the BMI distribution while holding the shape of the distribution constant at the reference year. The redistribution component represents the change in obesity as a result of a redistribution in the BMI curve while the mean BMI is kept constant. A third component, by definition, is the residual that cannot be exclusively attributed to the previous two elements. When applied to our study, the obesity rate at time *t*, *Obs*_*t*_, can be represented as

1 $$ Ob{s}_t=Obs\left(T\Big|{\mu}_t;{L}_t\right), $$

where *Obs* denotes obesity prevalence, *T* is the obesity threshold (30 for class I obesity), *μ *is the mean BMI, and *L* is the Lorenz curve representing the CDF of the empirical probability distribution of BMI. Letting *t*_*n* − 1_ be the base year, changes in obesity prevalence between two time-periods can then be decomposed as

2 $$ Ob{s}_{t_n}-Ob{s}_{t_{n-1}}\kern0.5em =\kern1em G\left({t}_{n-1},{t}_n\right)+R\left({t}_{n-1},{t}_n\right)+\varepsilon \left({t}_{n-1},{t}_n\right), $$

where *G*(⋅), *R*(⋅), and *ε*(⋅) represent the growth, redistribution, and residual components, respectively. Specifically, the growth and redistribution terms were defined as

$G\equiv Obs\left(T\Big|{\mu}_{t_n};{L}_{t_{n-1}}\right)-Obs\left(T\Big|{\mu}_{t_{n-1}};{L}_{t_{n-1}}\right)$, and$R\equiv Obs\left(T\Big|{\mu}_{t_{n-1}};{L}_{t_n}\right)-Obs\left(T\Big|{\mu}_{t_{n-1}};{L}_{t_{n-1}}\right)$. That is, *G* was the change in obesity driven by overall growth in population weight while holding relative position fixed, and *R* was the observed variation in relative position with no growth in mean BMI.

In empirical studies, a residual term controls for mis-specified components in the decomposition analysis, which confound the interpretation of decomposition results, particularly when the residual term is relatively large [39]. A more desirable method would decompose changes in prevalence measures exactly into growth and redistribution factors without a residual term. In this context, a Shapley-value based decomposition approach which takes an equally weighted average of two decompositions, one at a reference point and the other at a later year, has been proposed [23, 24]:

3 $$ Ob{s}_{t_n}-Ob{s}_{t_{n-1}}\kern0.5em =\kern1em {G}^s\left({t}_{n-1},{t}_n\right)+{R}^s\left({t}_{n-1},{t}_n\right) $$

where *G*^*s*^ and *R*^*s*^ represent the Shapley value of growth and distribution components of changes in obesity prevalence, and are given by:

$$ \begin{array}{l}{G}^s\equiv \frac{1}{2}\left\{Obs\left(T\Big|{\mu}_{t_n};{L}_{t_{n-1}}\right)-Obs\left(T\Big|{\mu}_{t_{n-1}};{L}_{t_{n-1}}\right)\right\}+\frac{1}{2}\left\{Obs\left(T\Big|{\mu}_{t_n};{L}_{t_n}\right)-Obs\left(T\Big|{\mu}_{t_{n-1}};{L}_{t_n}\right)\right\}\\ {}{R}^s\equiv \frac{1}{2}\left\{Obs\left(T\Big|{\mu}_{t_{n-1}};{L}_{t_n}\right)-Obs\left(T\Big|{\mu}_{t_{n-1}};{L}_{t_{n-1}}\right)\right\}+\frac{1}{2}\left\{Obs\left(T\Big|{\mu}_{t_n};{L}_{t_n}\right)-Obs\left(T\Big|{\mu}_{t_n};{L}_{t_{n-1}}\right)\right\}\end{array}. $$

### Obesity inequality indices (Gini and Generalized Entropy)

While distributional dominance tests offer a partial ranking of BMI distributions, they do not measure cardinal differences between distributions and there are cases in which stochastic dominance cannot be determined. Decomposition analysis is also limited in that it relies heavily on the specific obesity threshold, *T*, to decompose the variation. In this section, we supplement our previous findings by summarizing obesity inequality into a univariate concentration index, the Gini coefficient, and track the cardinal growth of obesity inequality. Typically used to quantify income and wealth inequality, the Gini coefficient measures the statistical dispersion in a given distribution. It varies between 0, which reflects complete equality, and 1, which indicates complete inequality (one person has all the income or wealth, all others have none). Our approach closely followed Sahn where the Gini index was employed to track the obesity inequality in developing countries [22]. The Gini coefficient is computed as follows:

4 $$ Gin{i}_t=\frac{2}{\mu_t{N}_t^2}{\displaystyle \sum_{i=1}^{N_t}{r}_{it}{x}_{it}}-\frac{N_t+1}{N_t}, $$

where *N* is the sample size, *μ *denotes the mean BMI, *x*_*i*_ and *r*_*i*_ represent individual BMI and corresponding rank of the *i*^*th*^ observation in ascending order. Considering the sample size in our study, we referred to a computation-efficient formula, which approximates the Gini coefficient using a fast optimized algorithm [40].

In addition to the common Gini coefficient as a summary measure of inequality, generalized entropy (GE) inequality measures have been proposed. Compared to the Gini coefficient, which is more sensitive to variations around the mode of the distribution, the GE measures are more flexible allowing greater sensitivity away from the middle of the distribution [41]. This is an attractive feature given the focus of recent studies on the rise in morbid obesity at the upper tail of the BMI distribution and its contribution to the overall rise in obesity. The Generalized Entropy index can be expressed as:

5 $$ G{E}_t\left(\theta \right)=\frac{1}{\theta \left(\theta -1\right)}\left[\frac{1}{N_t}{\displaystyle \sum_{i=1}^{N_t}{\left(\frac{x_{it}}{\mu_t}\right)}^{\theta }-1}\right], $$

where *θ* is a scaling parameter that represents the weight given to distances between individuals' BMI at different parts of the BMI distribution. For *θ* = 1 we obtained the Theil index, which treats differences between individuals' BMI levels at different points of the BMI distribution equally. The variation at the left tail of the distribution is given more weight with parameter values smaller than 1, whereas larger parameter values give more weight to the upper tail. We set *θ* equal to 0 and 2 for robustness and for comparison with a previous study examining this issue [41].

With distributional dominance tests and decomposition analysis, we were unable to split the analytic sample by demographic category and explore whether the growing obesity inequality is due to changing characteristics of particular population segments, or due to a population-wide shift of the BMI distribution. In the analysis of inequality over time, it could be the case that a growing inequality is influenced by greater disparities among different segments of the population, or by variation in the distribution of BMI within each subpopulation (provided the relative weight of different groups in the total population does not change). The GE class of inequality measures can be decomposed into within- and between-group inequality such that

*GE*_*t*_(*θ*)=*GE*_*t*_(*θ*)_*within*_ + *GE*_*t*_(*θ*)_*between*_ [41, 42]. Specifically, *GE*_*t*_(*θ*)_*within*_ = ${\displaystyle \sum_j\left(\frac{BM{I}_{t,j}}{BM{I}_t}\right)G{E}_{t,j}}$ and *GE*_*t*_(*θ*)_*between*_ = ${\displaystyle \sum_j\left(\frac{BM{I}_{t,j}}{BM{I}_t}\right) \ln \left(\frac{BM{I}_{t,j}/BM{I}_t}{N_{t,j}/{N}_t}\right)}$

where *GE*_*t,j*_ and *BMI*_*t,j*_ denote the GE index and BMI of subgroup *j* at time *t*, respectively; *N*_*t,j*_ represents the number of respondents in subgroup *j* at time *t*; and *BMI*_*t*_ represents the BMI of the total population at time *t*. That is, the first term indicates the weighted sum of inequalities within groups, whereas the second term captures the proportion attributable to the heterogeneity in inequality across the groups. If the contribution of between-group inequalities to total obesity inequality is negligible, and the evolution of within-group inequality is comparable across groups, this indicates that worsening obesity inequality is not a result of changing demographic composition (even if the weight of different groups in the total population has indeed changed), but rather more of a population-wide experience.

## Results and discussion

**Fig. 1:**
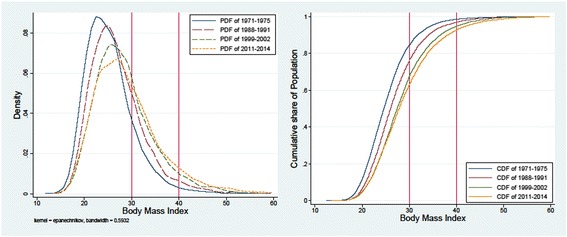
Distribution of BMI over time, 1971-2014

Studies reporting on the obesity epidemic have documented a dramatic increase in obesity prevalence over the last four decades [1–6]. In particular, the percentage increase in the higher obesity classes is substantial, suggesting that the population weight distribution has been disproportionately shifted rightward [11, 12, 43]. As illustrated in Fig. 1, which presents the kernel density and cumulative distribution of BMI over time, the increase in BMI was more pronounced between the late 1970s to early 2000s, whereas significant wave-to-wave differences have been small or not found in recent periods.

### Stochastic dominance tests

To better assess the long-term transition of US population weight, we present the results of SD tests on BMI distributions over time (Table 1). Unlike *t*-tests on prevalence estimates, stochastic dominance provides non-parametric pairwise comparisons of entire distributions (in our case at 30 points over the BMI domain) so that the comparisons of whether one CDF is greater in magnitude to the other can be made. Table 1 shows a clear pattern of BMI distribution dominance for all year-to-year comparisons up until the mid-2000s. Until the 2003-2006 survey, each BMI distribution first-order dominated the previous distribution, which indicates a significant difference for at least one test point without a significant crossing of distributions. That is, the temporal increase in cumulative distributions during this period was greater than or equal to zero over the domain of the BMI distribution.

**Table 1:** Stochastic dominance tests of the BMI distribution, 1971-2014

Survey years	Dominance tests
(1971-1974) - (1976-1980)	1
(1976-1980) - (1988-1991)	1
(1988-1991) - (1991-1994)	1
(1991-1994) - (1999-2002)	1
(1999-2002) - (2003-2006)	1
(2003-2006) - (2007-2010)	ND
(2007-2010) - (2011-2014)	−1
(1971-1974) - (1988-1991)	1
(1988-1991) - (1999-2002)	1
(1999-2002) - (2011-2014)	1

Note: 1 and ND represent first-order stochastic dominance and no dominance, respectively; -1 denotes that the 2007-2010 distribution dominates the 2011-2014. (No second or third order stochastic dominance were detected.) Data Source: NHANES

Although we observed significant temporal shifts in a statistical sense, the nature of the transition depends upon which part of the distribution most contributes to the dominance of one distribution over another. Table 2 shows t-statistics of first-order dominance tests at 30 test points covering the whole range of the BMI distributions. For instance, the first column compares the CDF of the 1976-1980 survey to the previous period, and dominance is declared only at the second test point. Although we rejected the null hypothesis of non-dominance at the first-order of comparison, this difference did not seem meaningful in an economically significant sense. From 1976-1980 through 1999-2002, a significant increase was observed across most of the domain, indicating population-wide upward shifts of the BMI distribution. More specifically, from 1976-1980 to 1988-1991 and 1999-2002 to 2003-2006, a significant increase was found at the very upper tail of the distribution (at the 29^th^ and 30^th^ test points), indicating an even more disproportionate shift than in other periods. The shift of the BMI distribution across 1988-1991 and 1991-1994 and 1999-2002 and 2003-2006 was relatively more pronounced around the middle of distribution, although the distribution was becoming more skewed in the latter period. The null hypothesis of non-dominance was not rejected during the 2003-2006 through 2007-2010 while the most recent comparison between 2007-2010 and 2011-2014 found a disproportionate downward shift across the very top end of the distribution.

**Table 2:** Significance test results for first-order stochastic dominance

	(1971-1974)-(1976-1980)	(1976-1980)-(1988-1991)	(1988-1991)-(1991-1994)	(1991-1994)-(1999-2002)	(1999-2002)-(2003-2006)	(2003-2006)-(2007-2010)	(2007-2010)-(2011-2014)
1	1.23	1.63*	0.90	1.30*	0.27	0.45	0.19
2	2.77***	1.79**	0.39	2.56***	0.38	0.03	−0.73
3	1.84**	4.45***	−0.51	3.86***	0.96	0.05	−1.71**
4	1.50*	4.08***	0.11	4.48***	0.77	0.20	−1.90**
5	0.89	5.50***	1.63*	5.75***	2.35***	0.25	−1.19
6	0.17	5.09***	2.35***	4.83***	1.85**	0.79	−0.74
7	0.07	4.63***	3.75***	6.47***	1.57*	1.19	−1.10
8	−0.50	4.60***	3.97***	6.92***	1.57*	1.20	−0.94
9	−1.16	6.55***	3.45***	7.54***	1.83**	1.48*	−0.94
10	−0.95	6.71***	3.01***	7.91***	1.90**	1.48*	−0.91
11	0.27	6.62***	2.39***	6.27***	2.12**	0.91	−1.12
12	0.73	6.80***	2.98***	4.76***	2.49***	0.90	−1.49*
13	0.25	5.45***	2.38***	5.26***	1.95**	1.03	−1.34*
14	0.23	5.61***	2.42***	5.23***	1.48*	1.14	−1.34*
15	0.73	5.35***	2.90***	5.07***	1.40*	1.12	−1.60*
16	0.81	4.56***	3.25***	4.43***	1.27	1.35*	−1.07
17	1.05	4.61***	3.06***	3.84***	1.27	1.56*	−0.96
18	0.46	4.99***	2.97***	4.12***	1.30*	1.70**	−1.17
19	0.42	4.47***	2.55***	3.69***	1.11	0.97	−1.43*
20	−0.13	4.05***	2.21**	3.31***	0.67	1.18	−2.04**
21	−0.49	3.80***	2.16**	3.40***	0.96	1.01	−1.77**
22	−0.48	3.93***	1.60*	3.71***	0.83	0.90	−1.90**
23	−0.90	4.51***	0.71	3.26***	1.11	0.72	−2.20**
24	−0.78	4.08***	0.74	2.63***	1.00	0.51	−2.24**
25	−0.88	3.50***	1.06	2.87***	0.72	0.16	−2.31**
26	−1.15	3.66***	0.84	2.63***	0.46	0.49	−2.09**
27	−1.00	2.72***	0.95	2.21**	0.24	0.27	−2.79***
28	−0.10	1.82**	0.97	1.38*	1.31*	−0.25	−2.20**
29	−0.91	2.50***	0.82	0.67	2.02**	−0.60	−2.77***
30	−0.89	2.91***	0.13	0.84	2.05**	−0.40	−2.29**

Note: *, **, and *** indicate *t*-test for difference at test point is statistically significant at 10, 5 and 1% level, respectively

Overall, results from the SD tests indicated that the distribution of BMI has disproportionately shifted upwards between 1971 and 2003, but this shift stalled in the mid-2000s.

### Growth-inequality decomposition

**Fig. 2:**
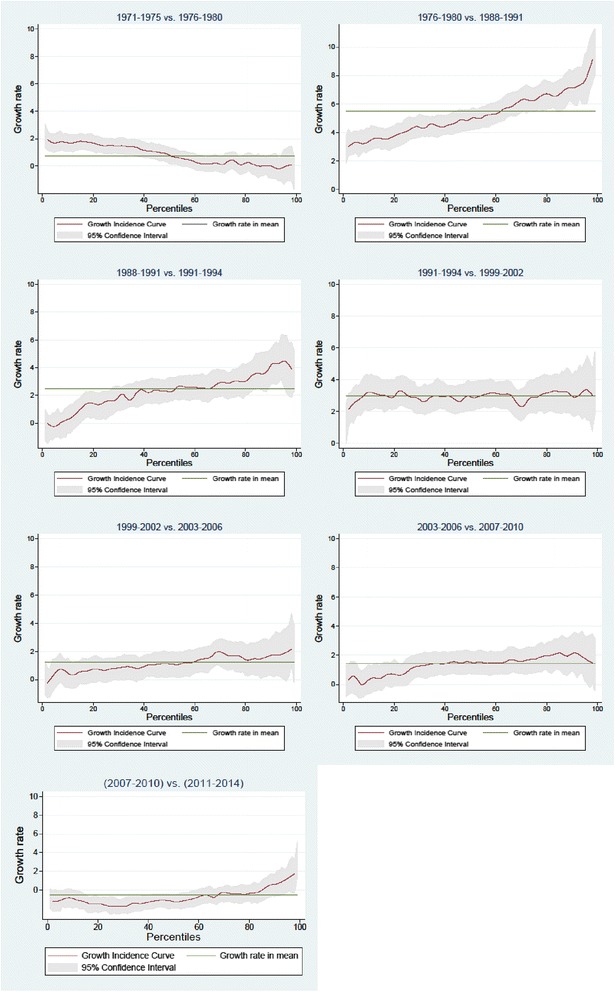
BMI growth curves

In addition to dominance test results, Growth Incidence Curves graphically describe which part of the BMI distribution contributed more to the overall growth between two sampling periods (Fig. 2). They show the percentage change at each BMI percentile with reference to the horizontal line representing the rate of prevalence growth [44]. Consistent with the SD test results, Fig. 2 shows a moderate BMI increase from NHANES I (1971-1974) to II (1976-1980) caused by the shift of the lower tail of the distribution. Since then, we observe a clear pattern of a rapid rise in obesity prevalence between 1976-1980 and 1991-1994 that was most pronounced in higher obesity percentiles. Interestingly, over 1991-1994 and 1999-2002 when the highest increase was experienced, the rate of obesity growth was approximately the same across all BMI levels.

Table 3 presents the results of the Shapley decomposition, which decomposed the total change in the obesity rate between sampling periods into growth and redistribution components. Not surprisingly, our results supported the SD test results in Table 2. For instance, between 1976-1980 and 1988-1991 when we observed an upward shift at the right tail of distribution, approximately 26.6 % of the increase in obesity prevalence was explained by the redistribution component. Through 1988-1991 and 1999-2002, the BMI distribution of subsequent periods first-order dominated the previous distribution mostly around the middle of the domain, and this was reflected in a smaller redistribution effect in the corresponding time frame. Similarly, the disproportionate shift from 1999-2002 to 2003-2006 being more pronounced at the right-tail was reaffirmed by a sizable redistribution effect in Table 3. Throughout the decomposition analysis, two clear results stand out. First, except for the initial sample period comparison of 1971-1975 to 1976-1980, the increase in obesity in the US has been predominately due to the mean-growth effect. This implies that the recent rise in obesity in the US has not been a statistical artifact applicable only to a particular population group or due to the arbitrariness of the obesity threshold. However, while playing a lesser role, the redistribution component was positive and non-trivial up until the last sampling period. This indicates that between the 1970s and 2000s there was a continual increase in obesity inequality. Second, an interesting change in dynamics was observed between 2003 and 2012. During this time span, when the change in the obesity rate slowed substantially compared to the previous two decades, the contribution of the redistribution component shrank substantially and became negative between 2007-2010 and 2011-2014. If this trend continues, this could indicate that the rise in obesity inequality observed in the previous three decades could be stalling or even reversing despite continued increases in obesity prevalence.

**Table 3:** Shapley decomposition of increase in obesity prevalence

Year	∆ in obesity prevalence	Growth component (A)	Redistribution component (B)	A/(A + B) (%)	B/(A + B) (%)
(1971-1975) - (1976-1980)	0.122	0.020	0.101	(16.68)	(83.32)
(1976-1980) - (1988-1991)	6.434	4.724	1.710	(73.42)	(26.58)
(1988-1991) - (1991-1994)	3.364	3.054	0.310	(90.78)	(9.22)
(1991-1994) - (1999-2002)	6.536	5.933	0.603	(90.77)	(9.23)
(1999-2002) - (2003-2006)	3.205	2.350	0.856	(73.30)	(26.69)
(2003-2006) - (2007-2010)	1.491	1.455	0.036	(97.55)	(2.44)
(2007-2010) - (2011-2014)	1.796	1.835	−0.040	(102.23)	(-2.23)

### Obesity inequality indices

Table 4 shows the historical trends for two different indices of BMI inequality: the Gini coefficient and Generalized Entropy when *θ* = 0 or 2. According to the Gini coefficient, there has been a steady and significant increase in obesity inequality in the US since the 1970s. Specifically, the degree of inequality measured by the Gini index increased most rapidly from the 1976-1980 to the 1988-1991 waves, followed by a relatively moderate but significant rise until 1999-2002. This pattern of transition is consistent with the SD test where first-order dominance was observed at the upper tail of distribution between 1976-1980 and 1988-1991. Similarly, the increase in obesity inequality from 1999-2002 to 2003-2006 was slightly greater than that of more recent periods as the top end of distribution significantly shifted.

**Table 4:** Intertemporal trends in obesity inequality

Year	Gini index	% Change from t-1 to t	95 % C.I.	Sensitivity Analysis	
				GE(2)	GE(0)
1971-1974	0.1040 (.0011)		(0.1019,0.1061)	0.0190	0.0174
1976-1980	0.1029*** (.0009)	−1.10	(0.1011,0.1047)	0.0185	0.0169
1988-1991	0.1117*** (.0015)	8.57	(0.1087,0.1147)	0.0221	0.0199
1991-1994	0.1166*** (.0018)	4.36	(0.1130,0.1201)	0.0240	0.0216
1999-2002	0.1221*** (.0014)	4.75	(0.1194,0.1248)	0.0257	0.0236
2003-2006	0.1242*** (.0013)	1.73	(0.1216,0.1268)	0.0271	0.0244
2007-2010	0.1260*** (.0012)	1.47	(0.1237,0.1284)	0.0277	0.0252
2011-2014	0.1282*** (.0013)	1.70	(0.1256,0.1308)	0.0286	0.0260
% Increase	23.27			50.47	49.31

Note: *** indicates *t*-test for difference in inequality index from t-1 to t is statistically significant at 1 % level. Jackknife standard errors in parentheses

Mirroring the results of the Gini coefficient, the Generalized Entropy index also indicated that obesity inequality increased significantly, but suggested that the rate of growth was even greater (about twice that of the Gini index). The growing obesity inequality measured by GE(2) and GE(0) corresponded closely, indicating the robustness of our findings regardless of the relative importance of the lower or upper tails of the distribution. Overall, our analysis of obesity inequality suggests that the US adult population has experienced growing obesity inequality.

**Fig. 3:**
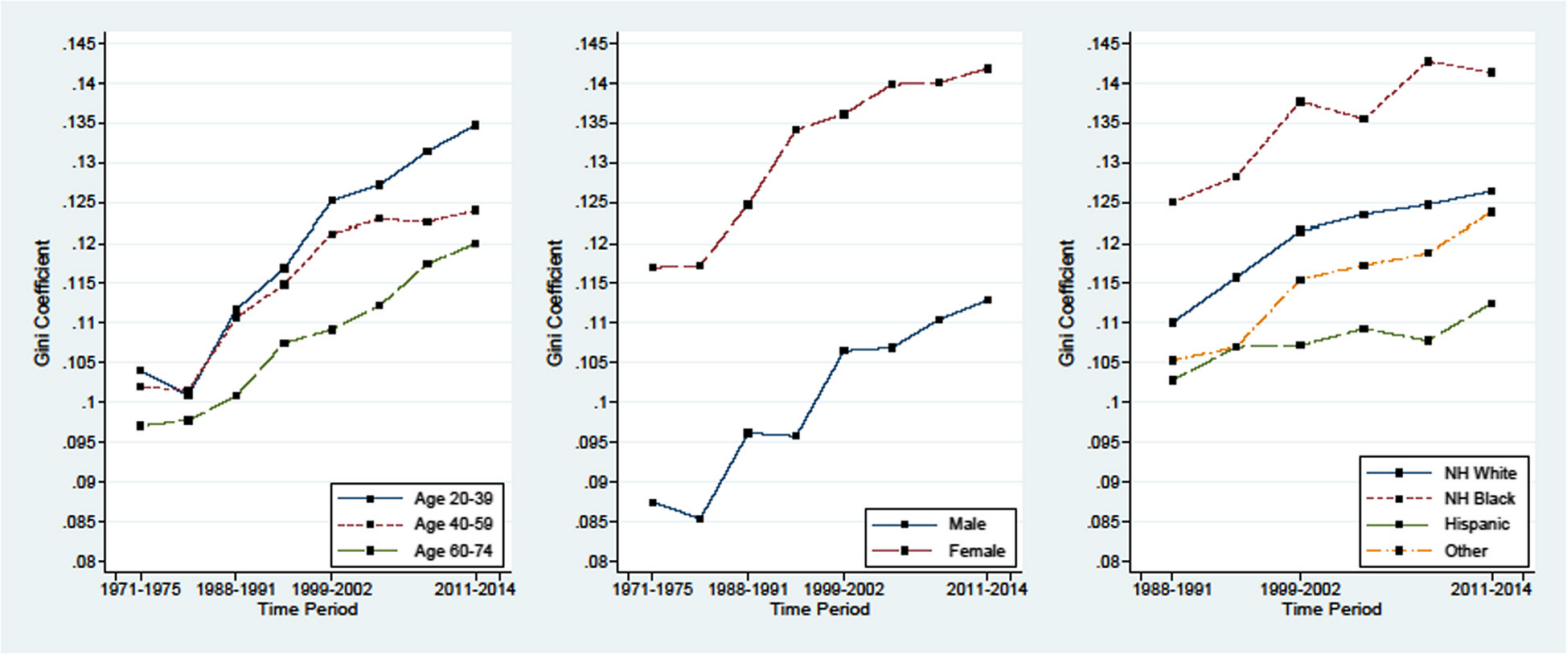
Trends in Gini coefficient by age, sex, and race

Breaking down the Gini coefficient based upon age, gender, and race categories indicates that the growth in obesity inequality has been a population-wide phenomenon across subpopulations (Fig. 3). For instance, both males and females have experienced a substantial increase in obesity inequality, with the rate of growth being nearly identical, although females started from a higher level of inequality. Consistent with Flegal and Troiano, we also found evidence of a more disproportionate shift of BMI distribution among younger adults [12]. We found no evidence that this disproportionate growth is due to particular ethnic groups, although the increase was less pronounced among the Hispanics.

Table 5 reports the obesity inequality decomposed into the within- and between-group component. Given the little difference between GE(0) and GE(2), we present a decomposition of GE(1) (i.e., the Theil index) by age, gender, race and combinations of these categories. As expected, most obesity inequality was due to the inequality within groups, and this pattern did not vary significantly over time. The between-group inequality was very small. For instance, approximately 0.66 % (=0.00016/0.02429) - 2.09 (=0.00056/0.02685) of total obesity inequality was attributable to race/ethnicity. Gender accounted for approximately 0.01-0.33 % of total obesity inequality, whereas age explained about 0.93-3.49 % of total obesity inequality. The combination of age, gender, and race accounted for only 3.38-4.76 % of inequality. More importantly, we did not find a systematic increase or decrease in the obesity inequality attributable to particular between-group components, although disparities among the ethnic groups appear to have increased slightly since 1999-2002. That is, this unequal shift of the BMI distribution has been more of a population-wide experience across the US.

**Table 5:** Within-group and between-group obesity inequality, Generalized Entropy (1)

Year	GE(1)		Age	Sex	Race	Age/Race	Sex/Race	Age/Sex/Race
(1971-1974)	0.01797	Within	0.01747 (97.22)	0.01791 (99.67)				
		Between	0.00050 (2.78)	0.00006 (0.33)				
(1976-1980)	0.0175	Within	0.01689 (96.51)	0.01748 (99.83)				
		Between	0.00061 (3.49)	0.00003 (0.17)				
(1988-1991)	0.0207	Within	0.02012 (97.20)	0.02069 (99.95)	0.02051 (99.08)	0.01987 (95.99)	0.02039 (98.50)	0.01979 (95.60)
		Between	0.00058 (2.80)	0.00001 (0.05)	0.00019 (0.92)	0.00083 (4.01)	0.00031 (1.50)	0.00091 (4.40)
(1991-1994)	0.02247	Within	0.02189 (97.42)	0.02247 (99.99)	0.02227 (99.11)	0.0216 (96.13)	0.02204 (98.04)	0.0214 (95.24)
		Between	0.00058 (2.58)	0.00000 (0.01)	0.0002 (0.89)	0.00087 (3.87)	0.00044 (1.96)	0.00107 (4.76)
(1999-2002)	0.02429	Within	0.02393 (98.52)	0.02426 (99.88)	0.02412 (99.34)	0.0237 (97.61)	0.02386 (98.27)	0.02347 (96.62)
		Between	0.00036 (1.48)	0.00003 (0.12)	0.00016 (0.66)	0.00058 (2.39)	0.00042 (1.73)	0.00082 (3.38)
(2003-2006)	0.02531	Within	0.02488 (98.30)	0.02531 (99.99)	0.02494 (98.54)	0.02443 (96.52)	0.02475 (97.79)	0.02424 (95.73)
		Between	0.00043 (1.70)	0.00000 (0.01)	0.00037 (1.46)	0.00088 (3.48)	0.00056 (2.21)	0.00108 (4.27)
(2007-2010)	0.02599	Within	0.02574 (99.04)	0.02599 (99.99)	0.02553 (98.23)	0.02523 (97.08)	0.02534 (97.50)	0.02495 (96.00)
		Between	0.00025 (0.96)	0.00000 (0.01)	0.00046 (1.77)	0.00076 (2.92)	0.00065 (2.50)	0.00104 (4.00)
(2011-2014)	0.02685	Within	0.02660 (99.07)	0.02680 (99.81)	0.02629 (97.91)	0.02599 (96.80)	0.02606 (97.06)	0.02571 (95.75)
		Between	0.00025 (0.93)	0.00005 (0.19)	0.00056 (2.09)	0.00086 (3.20)	0.00079 (2.94)	0.00114 (4.25)

Note: Age: 20-39, 40-59, 60-74; Sex: Male, Female, Race: non-Hispanic White, non-Hispanic Black, Hispanic and Other

Obesity inequality is not decomposed by race in (1971-1974) and (1976-1980) due to a lack of consistent definition of race category in NHANES

Within- and between-group inequality as a proportion of total inequality in parentheses

## Conclusions

Contributing to the growing literature focusing on different forms of inequality (e.g., income, wealth), this study quantifies through three alternative, complementary methods commonly employed in the economics literature, the trends in obesity inequality that have been experienced in the US during the past four decades. The methods range from ordinal comparisons of BMI distributions (through non-parametric tests of stochastic dominance between BMI distributions), to cardinal comparisons of inequality summarized in Gini and Generalized Entropy indices, including a decomposition of changes in the BMI distribution into mean growth, and a redistribution component driven by changes in the shape of the distribution. We used BMI data from the representative sample of adults in the NHANES (1971-2014), and found evidence consistent across the three methods that the rapid growth in obesity prevalence in the US has been accompanied by growing obesity inequality. Further, we found that a disproportionate shift of BMI distribution occurred when US obesity was increasing the most. This growth in inequality, which is not simply contained to an expansion in the right-tail of the BMI distribution of extremely morbidly obese adults, is found across the distribution of BMI and population subgroups.

While the increase in obesity rates in the US is a clear economic and medical concern due to the direct linkages between obesity and chronic illnesses and increased medical costs, the increase in obesity inequality is a problem as well for two key reasons: First, there is evidence that life satisfaction is influenced by the prevalence of obesity surrounding an individual [18]. This suggests that as obesity inequality expands, thus driving a greater wedge between individuals lower versus higher on the BMI distribution of society, the negative influence of obesity on life satisfaction could increase. Thus, the growth in obesity inequality raises the specter that the wide array of negative social and psychological consequences that have been linked to individual obesity levels could be exacerbated in an increasingly obesity-unequal nation. Identified consequences of obesity such as discrimination [45–48], depression [49–53], and increased social stigma [54], could potentially be intensified with growing obesity inequality. Second, because being obese may be easier in a fatter society as individuals judge their BMI relative to their peers, a more unequal BMI distribution may lead to further growth in obesity prevalence. A relaxation in relative weight standards may create a vicious circle, which imposes additional social costs on heavier societies. Overall, it is hoped that the presented measurements of obesity inequality will help spur further research to better understand not only the consequences of increased obesity prevalence but also how increased obesity inequality is affecting individuals across the BMI spectrum and society as a whole.

## Abbreviations

BMI: body mass index
NHANES: National Health and Nutrition Examination Survey
SD: stochastic dominance
CPS: Current Population Survey
CDF: cumulative distribution function

## References

[1] Flegal KM, Carroll MD, Kuczmarski RJ, Johnson CL (1998). Overweight and obesity in the United States: Prevalence and trends, 1960-1994. Int J Obes Relat Metab Disord.

[2] Flegal KM, Carroll MD, Ogden CL, Curtin LR (2010). Prevalence and trends in obesity among US adults, 1999-2008. JAMA.

[3] Kuczmarski RJ, Flegal KM, Campbell SM, Johnson CL (1994). Increasing prevalence of overweight among US adults: The National Health and Nutrition Examination Surveys, 1960 to 1991. JAMA.

[4] Ogden CL, Carroll MD (2010). Prevalence of overweight, obesity, and extreme obesity among adults: United States, trends 1960–1962 through 2007–2008. National Cent Health Stat.

[5] Ogden CL, Carroll MD, Kit BK, Flegal KM (2014). Prevalence of childhood and adult obesity in the United States, 2011-2012. JAMA.

[6] Wang Y, Beydoun MA (2007). The obesity epidemic in the United States - gender, age, socioeconomic, racial/ethnic, and geographic characteristics: A systematic review and meta-regression analysis. Epidemiol Rev.

[7] Christakis NA, Fowler JH (2007). The spread of obesity in a large social network over 32 years. N Engl J Med.

[8] Ogden CL, Carroll MD, Flegal KM (2008). High body mass index for age among US children and adolescents, 2003-2006. JAMA.

[9] Skelton JA, Cook SR, Auinger P, Klein JD, Barlow SE (2009). Prevalence and trends of severe obesity among US children and adolescents. Acad Pediatr.

[10] Sturm R (2003). Increases in clinically severe obesity in the United States, 1986-2000. Arch Intern Med.

[11] Sturm R (2007). Increases in morbid obesity in the USA: 2000–2005. Public Health.

[12] Flegal KM, Troiano RP (2000). Changes in the distribution of body mass index of adults and children in the US population. Int J Obes Relat Metab Disord.

[13] Burke MA, Heiland F (2007). Social dynamics of obesity. Econ Inq.

[14] Oswald AJ, Powdthavee N (2007). Book review feature: Two reviews of the challenge of affulence: Self‐control and well‐being in the United States and Britain since 1950. Econ J.

[15] Etilé F (2007). Social norms, ideal body weight and food attitudes. Health Econ.

[16] Dragone D, Savorelli L (2012). Thinness and obesity: A model of food consumption, health concerns, and social pressure. J Health Econ.

[17] Blanchflower DG, Landeghem B, Oswald AJ (2009). Imitative obesity and relative utility. J Eur Econ Assoc.

[18] Wadsworth T, Pendergast PM (2014). Obesity (sometimes) matters: The importance of context in the relationship between obesity and life satisfaction. J Health Soc Behav.

[19] Contoyannis P, Wildman J (2007). Using relative distributions to investigate the body mass index in England and Canada. Health Econ.

[20] Houle BC. Measuring distributional inequality: Relative Body Mass Index distributions by gender, race/ethnicity, and education, United States (1999–2006). J Obes. 2010;959658. 10.1155/2010/959658 PMC3065007 21461393

[21] Madden D (2012). A profile of Obesity in Ireland, 2002–2007. J R Stat Soc Series A.

[22] Sahn DE (2009). Weights on the rise: Where and for whom?. J Econ Inequal.

[23] Kolenikov S, Shorrocks A (2005). A decomposition analysis of regional poverty in Russia. Rev Dev Econ.

[24] Shorrocks AF (2013). Decomposition procedures for distributional analysis: A unified framework based on the Shapley value. J Econ Inequal.

[25] Deurenberg P (2001). Universal cut-off BMI points for obesity are not appropriate. Br J Nutr.

[26] Jolliffe D. The income gradient and distribution-sensitive measures of overweight in the US. National Poverty Center Working Paper Series 07-27 [http://ageconsearch.umn.edu/bitstream/25677/1/cp060622.pdf]. Accessed 26 Nov 2013.

[27] Burkhauser RV, Beyond CJ, BMI (2008). The value of more accurate measures of fatness and obesity in social science research. J Health Econ.

[28] Ezzati M, Martin H, Skjold S, Vander Hoorn S, Murray CJ (2006). Trends in national and state-level obesity in the USA after correction for self-report bias: Analysis of health surveys. J R Soc Med.

[29] Gorber SC, Tremblay M, Moher D, Gorber B (2007). A comparison of direct vs. self‐report measures for assessing height, weight and body mass index: A systematic review. Obes Rev.

[30] Johnson CL, Paulose-Ram R, Ogden CL, Carroll MD, Kruszan-Moran D, Dohrmann SM (2013). National health and nutrition examination survey. Analytic guidelines, 1999-2010. Vital Health Stat.

[31] National Center for Health Statistics. The Third National Health and Nutrition Examination Survey, NHANES III (1988–1994). http://www.cdc.gov/nchs/nhanes/nh3data.htm. Accessed on Feb 01 2016.

[32] Mohadjer L, Montaquila J, Waksberg J, Bell B, James P, Flores-Cervantes I, Montes M (1996). National Health and Nutrition Examination Survey III, Weighting and Estimation Methodology.

[33] Davidson R, Duclos JY (2000). Statistical inference for stochastic dominance and for the measurement of poverty and inequality. Econometrica.

[34] Anderson G (1996). Nonparametric tests of stochastic dominance in income distributions. Econometrica.

[35] Sahn DE, Stifel DC (2000). Poverty comparisons over time and across countries in Africa. World Dev.

[36] Sahn DE, Stifel DC (2002). Robust comparisons of malnutrition in developing countries. Am J Agr Econ.

[37] Foster JE, Shorrocks AF (1988). Poverty orderings. Econometrica.

[38] Sahn DE, Stifel DC, Younger SD. Inter-temporal changes in welfare: Preliminary results from nine African countries. CFNPP Working Paper No. 94 [http://pdf.usaid.gov/pdf_docs/pnacm776.pdf]. Accessed 01 Dec 2013.

[39] Datt G, Ravallion M (1992). Growth and redistribution components of changes in poverty measures: A decomposition with applications to Brazil and India in the 1980s. J Dev Econ.

[40] Karagiannis E, Kovacevic M (2000). A method to calculate the Jackknife variance estimator for the Gini coefficient. Oxford B Econ Stat.

[41] Sehili S, Elbasha EH, Moriarty DG, Zack MM (2005). Inequalities in self‐reported physical health in the United States, 1993‐1999. Health Econ.

[42] Shorrocks AF (1984). Inequality decomposition by population subgroups. Econometrica.

[43] Ruhm CJ (2007). Current and future prevalence of obesity and severe obesity in the United States. Forum Health Econ Pol.

[44] Ravallion M, Chen S (2003). Measuring pro-poor growth. Econ Lett.

[45] Frieze IH, Olson JE, Good DC (1990). Perceived and actual discrimination in the salaries of male and female managers. J Appl Psychol.

[46] Pingitore R, Dugoni BL, Tindale RS, Spring B (1994). Bias against overweight job applicants in a simulated employment interview. J Appl Psychol.

[47] Puhl R, Brownell KD (2001). Bias, discrimination, and obesity. Obes Res.

[48] Roehling MV (1999). Weight‐based discrimination in employment: Psychological and legal aspects. Pers Psychol.

[49] Carpenter KM, Hasin DS, Allison DB, Faith MS (2000). Relationships between obesity and DSM-IV major depressive disorder, suicide ideation, and suicide attempts: Results from a general population study. Am J Public Health.

[50] Friedman MA, Brownell KD (1995). Psychological correlates of obesity: Moving to the next research generation. Psychol Bull.

[51] Istvan J, Zavela K, Weidner G (1992). Body weight and psychological distress in NHANES I. Int J Obes Relat Metab Disord.

[52] Onyike CU, Crum RM, Lee HB, Lyketsos CG, Eaton WW (2003). Is obesity associated with major depression? Results from the Third National Health and Nutrition Examination Survey. Am J Epidemiol.

[53] Radloff LS (1977). The CES-D scale a self-report depression scale for research in the general population. Appl Psych Meas.

[54] Wang SS, Brownell KD, Wadden TA (2004). The influence of the stigma of obesity on overweight individuals. Int J Obes.

